# Jump-Chain Simulation of Markov Substitution Processes Over Phylogenies

**DOI:** 10.1007/s00239-022-10058-0

**Published:** 2022-06-02

**Authors:** Simon Laurin-Lemay, Kassandra Dickson, Nicolas Rodrigue

**Affiliations:** 1 Department of Biology, Carleton University, 209 Nesbitt Biology Building, 1125 Colonel By Drive, Ottawa, ON K1S 5B6, Canada; 2 Institute of Biochemistry, Carleton University, Ottawa, ON Canada; 3 School of Mathematics and Statistics, Carleton University, Ottawa, ON Canada

**Keywords:** Positive selection, CpG hypermutability, Substitution models, Site-interdependent models, Model violations, Likelihood ratio test, Approximate Bayesian Computation

## Abstract

We draw attention to an under-appreciated simulation method for generating artificial data in a phylogenetic context. The approach, which we refer to as jump-chain simulation, can invoke rich models of molecular evolution having intractable likelihood functions. As an example, we simulate data under a context-dependent model allowing for CpG hypermutability and show how such a feature can mislead common codon models used for detecting positive selection. We discuss more generally how this method can serve to elucidate the ways by which currently used models for inference are susceptible to violations of their underlying assumptions. Finally, we show how the method could serve as an inference engine in the Approximate Bayesian Computation framework.

**Supplementary Information:**

The online version of this article (10.1007/s00239-022-10058-0) contains supplementary material, which is available to authorized users.

## Introduction

Model-based analyses of sets of homologous DNA and amino acid sequences have become routine practice in the study of molecular evolution. By definition, models of molecular evolution make simplifying assumptions about the underlying evolutionary process. However, relaxing the assumptions commonly adopted in model-based inferences can be technically challenging. For instance, relaxing the assumption of independence between sites can require elaborate nested Markov chain Monte Carlo (MCMC) approaches [e.g., (Robinson et al. 2003)] or Approximate Bayesian Computation (ABC) (Laurin-Lemay et al. 2018b). Indeed, we may still be years away from the development of inference-capable methods utilizing models that account for most of the understood factors at play in molecular evolution.

In the meantime, understanding the quantitative impacts of model violations on current widely adopted inference methods is crucial. For example, do codon models used to detect positive selection at the amino acid level actually detect such features, or are they being deceived by unaccounted determinants of the molecular evolutionary processes? The use of simulations, based on richer models than those used for inference, can shed light of these issues.

Traditionally, simulation of molecular evolution over phylogenetic trees is done by relying on the calculation of transition probabilities (in the stochastic process sense, rather than the biochemical sense) by exponentiation of a substitution rate matrix and drawing a state at a descendant node of a branch in proportion with these computed probabilities. This is the mode of operation of well-known simulation software, such as Seq-Gen (Rambaut and Grassly 1997) and Evolver from PAML (Yang 2007). However, conducting simulations on the basis of matrix exponentiation is limited to the models where such calculations are tractable, typically, the models that can be readily used for inference.

The traditional simulation approaches cannot be used for studying models where the rate at a particular codon site might be influenced by the codon states at other sites. In its most general form, such a model would operate in the sequence state space [see, e.g., (Robinson et al. 2003; Rodrigue et al. 2009)] and would require a rate matrix that is $61^N$ by $61^N$ (assuming a universal genetic code that prohibits stop codons), where *N* is the length of the codon sequence. With a typical protein of, say, 300 codons, it is not possible to perform any matrix algebra on the resulting $61^{300}$ by $61^{300}$ dimensional rate matrix. Thus, conducting simulations under this class of models requires an alternative approach.

## The Jump-Chain Method

The jump-chain method relies on generating full realizations of the substitution process along the branches of the phylogeny by drawing dwell times as well as the nature of all events. Such simulations are used in many fields to study stochastic processes (Çinlar 1975; Gillespie 1977). The approach requires no matrix algebra on a substitution rate matrix and thus enables simulations with more complex models, such as those where the state space is at the level of the entire sequence. Ultimately, under such a model, simulating any particular substitution event—including the dwell time to that event and the nature of the substitution itself—requires knowledge of the state of the entire coding sequence. Although applicable under any model, the jump-chain method is the only approach currently known that allows one to generate artificial data under models with dependence between sites. In a phylogenetic context, it operates with the following steps:

*Designate the location of the root of the tree* Any point along the tree is acceptable as a root, if dealing with a time-reversible Markov substitution process as we will do here. On the other hand, the substitution model used for simulating does not need to be time reversible; there is no such constraint in the jump-chain simulation theory, although in such cases the root location becomes meaningful.

*Draw a root state* This step, common to both traditional and jump-chain methods, consists of a draw from the stationary distribution of the Markov process. For traditional models, such as the general time-reversible model or its special cases, this amounts to a simple draw of a nucleotide based on the nucleotide frequency parameters. When simulating under a more complex model where the stationary distribution is intractable [e.g., (Robinson et al. 2003; Rodrigue et al. 2005)], one can first simulate a long series of substitution events along an artificial branch (using the steps explained below) in order to obtain an initial state from which to simulate over the phylogeny. Doing so will be equivalent to a draw from the intractable stationary distribution. Alternatively, one could be interested in studying the substitution process starting from a real sequence, which could serve as the root state.

*Draw a waiting (dwell) time* The simulation process bifurcates independently from the root node, each branch using the root state drawn in the previous step. Along one of these branches, a random variable is drawn from an exponential distribution parameterized by the rate away from the root state. Under traditional models, the rate away from a state is equal to the negative of the corresponding diagonal entry in the substitution rate matrix. Otherwise, the rate away from a state is calculated as the sum of rates in the substitution matrix to all directly accessible states; assuming a point-mutation process, whereby multi-nucleotide events are assigned a rate of 0, there are at most 9*N* accessible states. Denoting the rate away from a state as *R*, the dwell time *t* is given by computing the probability integral transform of an exponential distribution of rate *R* and setting $t=-ln(1-U)/R$, where *U* is a uniform random draw from the unit interval.

*Draw the next state* If the dwell time drawn in the previous step does not exceed the length of the branch along which we wish to simulate, the next state is drawn with a probability proportional to the rate to that state in the substitution matrix. This draw thus only requires the rates of the directly accessible next states (i.e., that imply a point mutation). Once drawn, this state becomes the reference for the next dwell time to be simulated.

*Set the descendant node* The previous two steps are repeated until the drawn dwell time brings the process beyond the end of the branch. The state at the descendant node is thus set as the last state drawn, and the procedure of the previous two steps then bifurcates independently, and so on until a dwell time is drawn beyond the length of each of the terminal branches of the tree, thereby producing the simulated alignment.

The only disadvantage is when simulating over trees with numerous long branches, which can amount to drawing numerous substitution events and thus becoming time consuming. On the other hand, detailed substitution histories can themselves become a subject of study [e.g., (Nielsen 2002; Bollback 2005)].

## A Practical Example

As an example, we simulated data under a codon substitution model that allows for context-dependent hypermutability. Specifically, we consider the case of a cytosine that is followed by a guanine (CpG) along a protein-coding DNA sequence. The cytosine in a CpG context is often methylated in mammalian genomes (Tweedie et al. 1997), which gives it a high propensity to mutate to thymine through spontaneous deamination (Bird 1980). Because CpG contexts can span two adjacent codons, the widely held assumption of independence between sites becomes invalid. The first step in constructing such a model is therefore to envision the substitution process directly in the space of all possible codon sequences of a given length. Following the usual practice of traditional codon models, we focus on a point mutation-based process, which means that rates are only assigned to events in which the initial and final states differ by one nucleotide; the rates between two sequences with multiple nucleotide differences are set to zero. For a given sequence, the rate away is the sum of rates to all nearest sequences. This latter property means that the rate away from any given sequence of length *N* codons involves less than 9*N* terms, since there are only 9 nearest neighbor states for each codon (with stops codons excluded from the state space, there are less than 9 on average). For simplicity, one can represent the basic idea of the model in a simple 61-by-61 rate matrix, but with the understanding that a site-dependent parameter is also invoked, which requires the knowledge of the states at adjacent codons. In other words, with the site-dependent parameter, the rate specified for a given event at a particular codon site can change as the states at neighboring sites change. Thus, the rate from one codon *i* to another *j* (which differ only by one nucleotide at position *c*) at a particular site is given by:

1 $$\begin{aligned} Q_{ij}={\left\{ \begin{array}{ll} \varphi _{j_c}, &{} {if\,syn.\ tr.,}\\ \varphi _{j_c}\kappa , &{} {if\,syn.\ ts.\ non-CpG,}\\ \varphi _{j_c}\kappa \lambda , &{} {if\,syn.\ ts.\ CpG,}\\ \varphi _{j_c}\omega , &{} {if\,non-syn.\ tr.,}\\ \varphi _{j_c}\kappa \omega , &{} {if\,non-syn.\ ts.\ non-CpG,}\\ \varphi _{j_c}\kappa \omega \lambda , &{} {if\,non-syn.\ ts.\ CpG,}\\ \end{array}\right. } \end{aligned}$$

where $\varphi _{j_c}$ is the frequency of the target nucleotide, $\kappa$ is the transition over transversion rate ratio, $\lambda$ modulates the CpG transition rate, and $\omega$ is the non-synonymous to synonymous rate ratio. For our simulations, we used the nucleotide-level parameter values, tree topology, and branch lengths obtained from running the classic version of this codon substitution model ($\lambda =1$) on a mammalian data sets taken from Laurin-Lemay et al. (2018a). We explored three different values for $\omega$: 0.2, 0.5, and 0.8. For each of these values, we simulated 100 replicates with $\lambda =1$, i.e., the classic codon model, 100 replicates with $\lambda =4$, and another 100 replicates with $\lambda =8$, the latter being a typical value observed on mammalian data. This experiment was replicated with 9 other parameter conditions (for a total of 10; see supplementary materials).

**Fig. 1:**
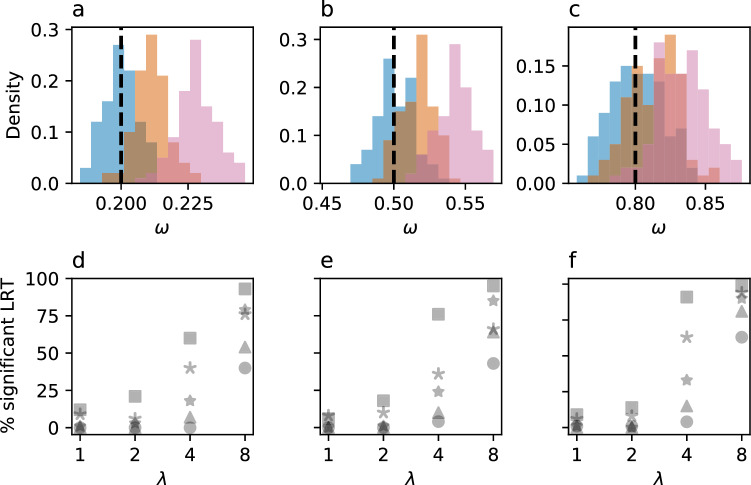
Distribution of maximum likelihood $\omega$ parameter values obtained from analyzing simulated alignments with M0 model from CodeML. Simulated alignments were generated under realistic conditions, corresponding to posterior distribution of M0 obtained from analyzing a mammalian alignment of the *WDR91* gene, with different $\omega _0$ values (black-dashed lines) and CpG transition rates (blue: $\lambda = 1$, orange: $\lambda = 4$, red: $\lambda = 8$). There were 100 replicates per condition. Details of the simulation grid are presented in supplementary materials. **a** All simulations are generated under $\omega _0 = 0.2$ (black-dashed line): 51%, 97%, and 100% of simulations had $\omega$ greater than the true value when $\lambda = 1$, $\lambda = 4$, and $\lambda = 8$, respectively. **b** All simulations are generated under $\omega = 0.5$ (black-dashed line): 50%, 90%, and 100% of simulations had $\omega$ greater than the true value when $\lambda = 1$, $\lambda = 4$, and $\lambda = 8$, respectively. **c** All simulations are generated under $\omega = 0.8$ (black-dashed line): 49%, 73%, and 95% of simulations had $\omega$ greater than the true value when $\lambda = 1$, $\lambda = 4$, and $\lambda = 8$, respectively. **d**–**f** Proportion of simulations (y-axis) rejecting the M7 model upon likelihood ratio test conducted with both M7 and M8 models (2 degrees of freedom). Simulated data where generated under 5 different mixtures of $\omega$ values with equally distributed values among sites from each mixture component, along with 4 levels of CpG transition rates. For realism, simulations were conducted using posterior average parameter values under M0 obtained by analyzing mammalian alignments of *STRIP1*, *GPAM*, and *WDR91* genes for panels d, e, and f, respectively. Circle, star, asterisk, triangle, and square markers correspond to $\omega$-mixture 1 (0.1, 0.2, 0.3), mixture 2 (0.4, 0.5, 0.6), mixture 3 (0.7, 0.8, 0.9), mixture 4 (0.2, 0.5, 0.7), and mixture 5 (0.5, 0.7, 0.9), respectively (Color figure online)

In panels a, b, and c of Fig. 1, we report the maximum likelihood values of $\omega$ obtained under the M0 model within CodeML (Yang 2007), with the distribution of all 100 values shown as a histogram. For the simulations with $\lambda =1$, the recovered $\omega$ values closely match those used for the simulations (marked with a dashed line), with about half of the simulations on either side of the true value. For simulations with $\lambda =4$, the M0 model overestimates $\omega$ values in most replicates. Nearly all $\omega$ values are overestimated in simulations with $\lambda =8$. These results suggest that applying the M0 model to data where CpGs are hypermutable is likely to lead to an overestimation of the key parameter of interest (see Supplementary Materials for details).

Panels d, e, and f of Fig. 1 show a similar set of experiments, but with data sets simulated with a mixture (equally-weighted) of $\omega$ values. For each set of 100 simulations with different $\lambda$ values, the panels indicate the percentage of significant likelihood ratio tests recovered from running M7 and M8 models within CodeML. In other words, the y-axis shows the percentage of replicates that reject the null M7 model, suggesting the presence of positive selection. At $\lambda =4$ and particularly at $\lambda =8$, a large proportion of simulations would be considered to contain signals of positive selection, although all simulations were conducted with mixtures of $\omega$ values less than 1. Again, these results suggest that such a classic statistical test with codon models is susceptible to error in the presence of CpG hypermutability (see Supplementary Materials for details).

## Future Uses

Phylogenetic simulations using the jump-chain method were used with a substitution model with dependence between sites by Robinson et al. (2003) as means of verifying their implementation. They have also been used as a means to performing posterior predictive checks [e.g., (Nielsen 2002; Rodrigue et al. 2006; Rodrigue et al. 2009; Lartillot et al. 2007; Laurin-Lemay et al. 2018b)]. In recent years, such simulations have been used to study the effect of epistasis on new models (Rodrigue and Lartillot 2017; Latrille et al. 2021). As done here, Laurin-Lemay et al. (2018a) explored the effect of CpG hypermutability on a test for detecting codon usage bias. Most of these applications remain within a small circle of researchers. We believe the method has an under-appreciated simplicity that is important to the study of much richer evolutionary models than those currently used for inference.

The method also has the potential to serve as a central kernel for the development of new inference-capable models that do not have a tractable closed-form likelihood, using ABC. The general idea of the ABC approach is to simulate a very large number of data sets, for instance, using the jump-chain method, utilizing a wide range of parameter values, and retaining the parameter values that produced data sets very similar to the true data set of interest. Indeed, Laurin-Lemay et al. (2018b) used this simulation approach in the context of an ABC implementation to study a site-dependent mutational process of CpG hypermutability within a mutation–selection framework.

**Fig. 2:**
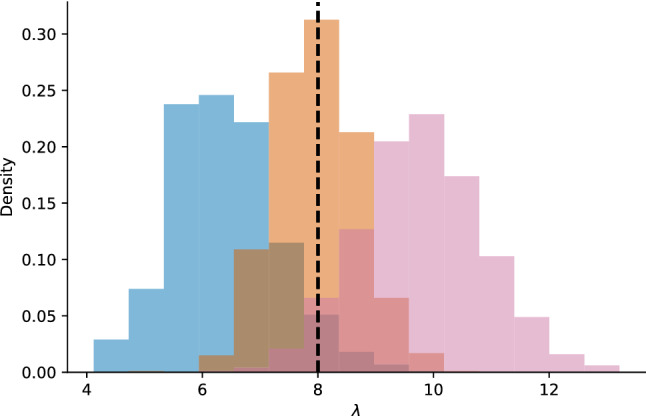
Posterior distribution of $\lambda$ recovered using CABC methodology when analyzing three simulated alignments (see Supplement Materials) generated with a CpG transition rate of $\lambda =8$. For one of the simulations (blue histogram), $\lambda$ has posterior mean of 6.38 with 95% credibility interval of 4.64–8.39. For a second simulation (red histogram), $\lambda$ has posterior mean of 9.78 with 95% credibility interval of 7.74–11.99. For a third simulation (orange histogram), $\lambda$ , and has a posterior mean of 7.99 with 95% credibility interval of 6.62–9.40 (Color figure online)

We applied the methods of Laurin-Lemay et al. (2018b) to simulations with $\lambda =8$ in the context of the more classical codon model studied here, to see if it could provide reasonable estimates of $\lambda$ (see Supplementary Materials for details). As a preliminary exploration and to keep calculations manageable, we applied the method to only three simulations. Note that the simulations are stochastic processes, which will vary from one instance to the next; the actual realized CpG hypermutability can be slightly higher, or lower, than the parameter value used for simulation. All three inferences yield posterior distributions for $\lambda$ that are close to the true value and include it within their 95% credibility intervals (Fig. 2). These preliminary results suggest that the method could be used not only to measure the level of CpG hypermutability but also to study the impact of accounting for this site-dependent process on other parameters, including those used to detect positive selection.

Altogether, we hope these short demonstrations will encourage other developers to explore the jump-chain simulation method within their work, either to study the robustness of their inferences to potential model violations or as a means of accounting for more of the complexities governing molecular evolution.

## Supplementary Information

Below is the link to the electronic supplementary material.

Supplementary file1 (PDF 199 kb)

Supplementary file2 (CSV 9 kb)

Supplementary file3 (CSV 20 kb)

Supplementary file4 (CSV 9 kb)

Supplementary file5 (CSV 90 kb)

Supplementary file6 (CSV 105 kb)

Supplementary file7 (CSV 11 kb)

Supplementary file8 (CSV 20 kb)

Supplementary file9 (CSV 35 kb)
