# Supplementary material for: Jump-Chain Simulation of Markov Substitution Processes Over Phylogenies
Source: J Mol Evol. 2022 Jun 2;90(3-4):239–43. doi: 10.1007/s00239-022-10058-0 (PMC9233627; doi:10.1007/s00239-022-10058-0)
Supplement: Supplementary file 1 — Supplementary file1 (PDF 199 kb) [file 239_2022_10058_MOESM1_ESM.pdf]

Supplement materials

Simon Laurin-Lemay[1], Kassandra Dickson[1], and Nicolas Rodrigue[1,2,3]

[1]Department of Biology, Carleton University, Ottawa, Canada
[2]Institute of Biochemistry, Carleton University, Ottawa, Canada
[3]School of Mathematics and Statistics, Carleton University, Ottawa, Canada

Contents

1 Datasets and Tree Topology **2**

2 Codon Substitution Models **2**

3 Simulation Study **3**
3.1 Detection of Background Purifying Selection with M0 Model . . 4
3.2 Detection of Positive Selection with M7 / M8 Models 4

4 Simulation-Based Inference **4**
4.1 Application of Conditional Approximate Bayesian Computation Using M0 Model 5

5 Tables **7**

1 Datasets and Tree Topology

From the 137 mammalian protein-coding gene alignments employed in Laurin-Lemay et al (2018b) we selected the same subset of genes, i.e., 10 genes, as used for validating the approach. The 10 genes were choosen for their GC3 content, ranging from $\sim 40\%$ to 75%. We also used the mammalian tree composed of 39 placentals species from that same publication Laurin-Lemay et al (2018b). Both the alignments and the phylogenetic tree are available via the GitHub repository `https://github.com/Simonll/jump-chain-simulation`; last accessed May 1, 2022).

2 Codon Substitution Models

In this work, we employed phylogenetic codon substitution models to explicitely test hypotheses about mutation and selection processes implemented in the the so-called mutation-selection framework (as reviewed in McCandlish and Stoltzfus, 2014; Teufel et al, 2018). The mutation-selection models used in this work differ in their definition of the mutation and/or selection processes. We used both site-independent and site-interdependent models. The site-independent models are implemented in three different sofware packages: PAML (inference and simulation: Yang, 2007), Phylobayes-MPI (inference and simulation: Lartillot et al, 2013) and our simulator (simulation: Laurin-Lemay et al, 2018a,b), while the site-interdependent codon substitution models are only implemented in our simulator.

All the models employed in this work use a primary site-independent mutation process parametrized with either the HKY or the GTR parameterization. The architecture of this primary mutation process is inspired from the original Muse and Gaut (1994) (MG) model and corresponds to the F1 x 4 definition made available in CodeML software from PAML (Yang, 2007). We explicitely chose the F1 x 4 architecture because the mutation process is more mechanisticly informed then its alternative Goldman and Yang (1994) (GY) as indicated in Rodrigue et al (2008).

The parameterizations of the mutation processes employed allow only point mutations, meaning that any substitutions between two codons, i and j, occure at a specific codon position, defined by the codon coordinates, $c = 0, 1, 2$. The two site-independent mutational parametrizations, HKY and GTR use 4 nucleotide propensities (3 degrees of freedom), with $\varphi = (\varphi_n)_{1 \leq n \leq 4}$, and with $\sum_{n=1}^{4} \varphi_n = 1$. We used the default settings of CodeML regarding the estimation of nucleotide propensities, i.e. calculating the frequency of each base from the analyzed alignment, which reduces the computation time. The HKY and the GTR parametrizations differ in the degree of heterogeneity they allow to model: the HKY parametrization allows transitions and transversions rates to differ with a single parameter, κ, whereas the GTR model allows a heterogeneous rate among all unordered single pairs of nucleotide (i.e., $A <> C$, $A <> G$, $A <> T$, $C <> G$, $C <> T$, $G <> T$) using 6 nucleotide exchangeabilities

(5 degrees of freedom) with $\varrho = (\varrho_{mn})_{1 \leq m,n \leq 4}$, and with $\sum_{1 \leq m < n \leq 4} \varrho_{mn} = 1$. The HKY model is nested within the GTR model, and thus we can modify the parameter values obtained under the GTR model to retrieve what would be the HKY equivalent. We also use a site-interdependent mutational process that together with the HKY model, allows us to model the CpG transition rate.

Stop codons are considered missing data if they are present in the alignments. All codon substitution models assume infinite purifying selection against stop codons, stop codons cannot be part of the substitution process proposed by any of the models used in this study. The simplest model, M0-HKY (M0) (Yang et al, 2000), uses the site-independent HKY mutational process and account for purifying selection on amino acids using one ω parameter as detailed in the following equation 1:

$$Q_{ij} = \begin{cases} \varphi_{j_c}, & \text{if syn. tv.,} \\ \varphi_{j_c}\kappa, & \text{if syn. ts.,} \\ \varphi_{j_c}\omega, & \text{if non-syn. tv.,} \\ \varphi_{j_c}\kappa\omega, & \text{if non-syn. ts.,} \end{cases} \tag{1}$$

where ≪syn.≫ and ≪non-syn.≫ are short for ≪synonymous≫ and ≪non-synonymous≫, ≪tv.≫ and ≪ts.≫ are short for ≪transversion≫ and ≪transition≫, and j_c returns the nucleotide found at the cth position of codon j. M0-GTR model only differ from previous model by its mutational parametrization using GTR instead of HKY paramterization. The M0-GTR model is detailed in equation 2:

$$Q_{ij} = \begin{cases} \varrho_{i_c j_c}\varphi_{j_c}, & \text{if syn.,} \\ \varrho_{i_c j_c}\varphi_{j_c}\omega, & \text{if non-syn.} \end{cases} \tag{2}$$

We also employed the M7-HKY (M7) and M8-HKY (M8) models (Yang et al, 2000), known as the sites models in CodeML (Yang, 2007). The M7 and M8 models are designed to detect positive selection by computing a likelihood ratio test, M7 model beeing nested within the M8 model. The M7 model is used to detect site-heterogeneous background purifying selection on amino acids ($\omega < 1$) as well as neutral evolution ($\omega \sim 1$) using a beta distribution defined on the unit interval. The beta distribution is hyperparametrized with, p and q, allowing the beta distribution to adopte different shapes: U-shaped, uniform, L-shaped distribution, etc. The beta distribution is then discretized into equal-sized intervals of K to allow for site heterogeneous purifying selection on amino acids (Yang, 1994). The median values of each interval define the K possible ω values. By definition, each ω value represents an equal proportion of sites $\frac{1}{K}$. The M8 model includes, in addition to the M7 model, a parameterization that allows to model the positive selection with the use of two parameters: one allowing $\omega > 1$, and another parameter to modulate the proportion of sites under positive selection.

3 Simulation Study

The objective of this simulation study is to evaluate how confounding factors at the mutational process level, namely a high rate of CpG transition (> 1), can affect the ability of (1) CodeML's M0 model to accurately detect a site-homogeneous background purifying selection on amino acids and the ability of (2) CodeML's M7 / M8 models to accurately detect sites under positive selection by calculating a likelihood ratio test.

3.1 Detection of Background Purifying Selection with M0 Model

We first evaluated the ability of M0 model from CodeML to detect background purifying selection on amino acids under 3 purifying selection regimes using different values of ω, i.e. 0.2, 0.5 and 0.8, and along 4 levels of CpG transition rate, $\lambda = 1, 2, 4, 8$. To ensure that simulation conditions are as realistic as possible, simulations were generated by sampling paramter values from posterior distributions previously obtained using M0-GTR implemented in Phylobayes-MPI (Lartillot et al, 2013), and by transforming the GTR paramterization within the HKY parametrization. Ten different mammalian gene alignments (see Datasets and Tree Topology) were analysed with M0-GTR model from Phylobayes-MPI. For each mammalian gene alignment, 100 replicates were drawn from the posterior distribution for simulation purpose, resulting in a total of 12,000 simulated alignments (10 genes $\times$ 3 purifying selection regimes $\times$ 4 levels of λ $\times$ 100 replicates) to be analysed with M0 model from CodeML. The test consists of computing $p(\omega > \omega_0)$, where ω_0 is the true value used for simulation purpose and ω is the maximum likelihood value recovered from analysing the simulated alignments.

3.2 Detection of Positive Selection with M7 / M8 Models

We secondly evaluate the ability of M7 / M8 models to detect adapative evolution in presence of CpG transition rate. We generated simulated alignments under the M7 model, where site-specific ω values are uniformly drawn from one of the 5 mixture of ω values (mixture 1: 0.1, 0.2, 0.3; mixture 2: 0.4, 0.5, 0.6; mixture 3: 0.7, 0.8, 0.9; mixture 4: 0.2, 0.5, 0.7, mixture 5: 0.5, 0.7, 0.9) and along 4 levels of CpG transition rate, $\lambda = 1, 2, 4, 8$. We evaluated the presence of positive selection by computing the likelihood ratio test (2 degrees of freedome) between M7 and M8 models for an alpha significance level of 5% using the chi square distribution. We calculated the poportion of significant tests across all replicates of each given condition, for a total of 20,000 simulated alignments (10 genes $\times$ 5 mixtures of purifying selection $\times$ 4 levels of λ $\times$ 100 replicates) to be analyzed with both M7 and M8 models.

4 Simulation-Based Inference

The most widely used ”likelihood-free inference method” or simulation based-inference is without question the Approximate Bayesian Computation (ABC) (Rubin, 1984; Beaumont et al, 2002), which is extensively used in population genetics (e.g. Wegmann et al, 2010). Simulation-based inference avoids likelihood calculation by using instead a collection of simulated data along with their parameter values. There are different algorithms for sampling the posterior distribution when using ABC: Rejection-Sampling, Sequential Monte Carlo (Toni et al, 2008), Markov Chain Monte Carlo (Marjoram et al, 2003), etc. Rejection-Sampling is the simplest ABC algorithm, where simulated data are generated using parameter values drawn from Bayesian model priors and accepted when the simulated data exactly matches the observed data. The match condition can be relaxed by introducing the use of summary statistics describing the data (observed data and simulated), and a metric to be computed over the vectors of summary statistics to assess the relative position of each simulation to the observed data. An acceptation threshold guaranteeing that the sampling of the parameter values operates from the posterior distribution can also be used. Utimately, we would like the summary statistics to not only be strongly correlated with the paramter values, but to be sufficient, meaning that these summary statistics carry all the information necessary to the conditioning of the parameters of the model when asymptotic conditions are reached (enough simulations are used). In practice, the summary statistics are generaly only strongly correlated to parameter values, and the acceptation threshold is set *a posteriori* by accepting a predefined proportion of the closest simulations to the observed data. This implies that the ABC methodology has been validated by computing the error and the coverage statistics of the model (Cook et al, 2006; Talts et al, 2020). The number of simulations to be performed as well as the quality of signal contained in the summary statistics, define the level of approximation that is made upon the simulation-base inference framework. Usually, selection and design of summary statistics is done on the basis of scientific knowledge. Several strategies employing regression model and preprocessing of summary statistics as well as paramter values are used to get even closer to the true posterior (e.g. Beaumont et al, 2002; Raynal et al, 2018).

4.1 Application of Conditional Approximate Bayesian Computation Using M0 Model

Conditional Approximate Bayesian Computation (CABC) is a variant of ABC, which was first applied in the mutation-selection framework in Laurin-Lemay et al (2018b), where the parameters of interest, such as the CpG transition rate, are conditioned given parameter values taken from another model previously conditioned in an exact Bayesian framework. In this work, we used the same inference conditions as used in Laurin-Lemay et al (2018b) to condition λ, but under a simpler model. We rather used the M0 model, which is not accounting for site-specific amino acids preferences and global deviation from

non-synonymous to synonymous neutral expectation using ω_* (Rodrigue and Lartillot, 2017). We apply the CABC methodology to the alignments previously simulated in this work (see Detection of Background Purifying Selection with M0 Model) with known parameter values, $\lambda = 8$ and $\omega = 0.5$, for the ten mammalian genes used in this study. The same prior were used as in (Laurin-Lemay et al, 2018b), excepted we used HKY parametrization instead of GTR, and by replacing λ_{ω_*}, with λ_ω as background purifying selection using site-specific amino acids preferences was not accounted for in M0 model. We used 10^5 simulations, while other conditions for CABC methodology were kept as in (Laurin-Lemay et al, 2018b). Finaly, to realisticlly display the level of stochasticity involves in the inference of λ parameter, we have arbitrarily chosen three posterior distributions: one almost centered on the true value, and two other posterior distributions of the same parameter, one underestimating the true value and the other overestimating it.

5 Tables

Table Sup1: Descriptive statistics of parameter value distributions used to generate simulated alignments under M0 model. The descriptive statistics from each row corresponds to an experiemental condition with the use of 100 replicates. We computed as descriptive statistics mean and 0.025-0.975 quantiles. Nucleotide propensities are denoted by `phi_A`, `phi_C`, `phi_G` and `phi_T`. Nucleotide exchangeabilities are denoted by `rho_AC`, `rho_AG`, `rho_AT`, `rho_CG`, `rho_CT` and `rho_TG`. Note that for each replicate: `rho_AC` = `rho_AT` = `rho_CG` = `rho_TG` and `rho_AG` = `rho_CT` by definition of HKY parametrization. Only one ω value was used per experimental condition (see mix column), which is confirmed by the proportion of sites under each possible ω values denoted by `omega_0.2`, `omega_0.5` and `omega_0.8`.

Table Sup2: Descriptive statistics of parameter value distributions used to generate simulated alignments under M7 model. The descriptive statistics from each row corresponds to an experiemental condition with the use of 100 replicates. We computed as descriptive statistics mean and 0.025-0.975 quantiles. Nucleotide propensities are denoted by `phi_A`, `phi_C`, `phi_G` and `phi_T`. Nucleotide exchangeabilities are denoted by `rho_AC`, `rho_AG`, `rho_AT`, `rho_CG`, `rho_CT` and `rho_TG`. Note that for each replicate: `rho_AC` = `rho_AT` = `rho_CG` = `rho_TG` and `rho_AG` = `rho_CT` by definition of HKY parametrization. Three ω values were used per experimental condition (see mix column), which is confirmed by the proportion of sites under each possible ω values denoted by ω values (`omega_0.1`, `omega_0.2`, `omega_0.3`, `omega_0.4`, `omega_0.5`, `omega_0.6`, `omega_0.7`, `omega_0.8` and `omega_0.9`).

Table Sup3: Maximum likelihood parameter values recovered under M0 model from CodeML when analysing simulated data generated under realistic conditions along 3 different values of ω $0.2, 0.5, 0.8$ and 4 levels of CpG transition rates,i.e., λ $1, 2, 4, 8$. See Table Sup1 for descriptive statistics of parameter value distributions used to generate simulated alignments under M0 model.

Table Sup4: Maximum likelihood parameter values recovered under M7 model from CodeML when analysing simulated data generated under realistic conditions along 5 different mixture of ω values and 4 levels of CpG transition rates,i.e., λ $1, 2, 4, 8$. See Table Sup2 for descriptive statistics of parameter value distributions used to generate simulated alignments under M7 model.

Table Sup5: Maximum likelihood parameter values recovered under M8 model from CodeML when analysing simulated data generated under realistic conditions along 5 different mixture of ω values and 4 levels of CpG transition rates,i.e., λ $1, 2, 4, 8$. See Table Sup2 for descriptive statistics of parameter value distributions used to generate simulated alignments under M7 model.

Table Sup6: Likelihood ratio test values recovered under M7 and M8 models from CodeML when analysing simulated data generated under realistic conditions along 5 different mixture of ω values and 4 levels of CpG transition rates, i.e., λ $1, 2, 4, 8$.

Table Sup7: Descriptive statistics of parameter value posterior distributions recovered from application of CABC to the M0 model. Each row corresponds to

an CABC analysis using a simulated alignment as ground true. The simulated alignments used as ground true were taken from previous simulations made under M0 model (Table Sup 1). row corresponds to descriptive statistics, mean and 95% credibility interval, computed from posterior distribution of following parameters: root, lambda, `lambda_TBL` (correction on tree length), `lambda_omega` (correction on ω), `phi_A`, `phi_C`, `phi_G`, `phi_T`, `rho_AC` and `rho_AC`.

Table Sup8: Descriptive statistics of parameter value posterior distributions obtained from M0GTR model over the 10 mammalian protein-coding gene alignments. We computed as descriptive statistics mean and 0.025-0.975 quantiles. Nucleotide propensities are denoted by `phi_A`, `phi_C`, `phi_G` and `phi_T`. Nucleotide exchangeabilities are denoted by `rho_AC`, `rho_AG`, `rho_AT`, `rho_CG`, `rho_CT` and `rho_TG`. Total tree length is denoted by `tree` and the nonsynonymous to synonymous subtitution rate parameter is denoted by `omega`.

References

Beaumont MA, Zhang W, Balding DJ (2002) Approximate bayesian computation in population genetics. Genetics 162(4):2025–2035

Cook SR, Gelman A, Rubin DB (2006) Validation of software for bayesian models using posterior quantiles. J Comput Graph Stat 15(3):675–692, DOI 10.1198/106186006x136976

Goldman N, Yang Z (1994) A codon-based model of nucleotide substitution for protein-coding DNA sequences. Mol Biol Evol 11(5):725–36, DOI 10.1093/oxfordjournals.molbev.a040153

Lartillot N, Rodrigue N, Stubbs D, Richer J (2013) PhyloBayes MPI: Phylogenetic reconstruction with infinite mixtures of profiles in a parallel environment. Syst Biol 62(4):611–615, DOI 10.1093/sysbio/syt022

Laurin-Lemay S, Philippe H, Rodrigue N (2018a) Multiple factors confounding phylogenetic detection of selection on codon usage. Mol Biol Evol 35(6):1463–1472, DOI 10.1093/molbev/msy047

Laurin-Lemay S, Rodrigue N, Lartillot N, Philippe H (2018b) Conditional approximate bayesian computation: A new approach for across-site dependency in high-dimensional mutation–selection models. Mol Biol Evol 35(11):2819–2834, DOI 10.1093/molbev/msy173

Marjoram P, Molitor J, Plagnol V, Tavare S (2003) Markov chain monte carlo without likelihoods. Proc Natl Acad Sci U S A 100(26):15,324–15,328, DOI 10.1073/pnas.0306899100

McCandlish DM, Stoltzfus A (2014) Modeling evolution using the probability of fixation: History and implications. Q Rev Biol 89(3):225–252, DOI 10.1086/677571

Muse S, Gaut B (1994) A likelihood approach for comparing synonymous and nonsynonymous nucleotide substitution rates, with application to the chloroplast genome. Mol Biol Evol 11(5), DOI 10.1093/oxfordjournals.molbev.a040152

Raynal L, Marin JM, Pudlo P, Ribatet M, Robert CP, Estoup A (2018) ABC random forests for bayesian parameter inference. Bioinformatics 35(10):1720–1728, DOI 10.1093/bioinformatics/bty867

Rodrigue N, Lartillot N (2017) Detecting adaptation in protein-coding genes using a bayesian site-heterogeneous mutation-selection codon substitution model. Mol Biol Evol 34(1):204–214, DOI 10.1093/molbev/msw220

Rodrigue N, Lartillot N, Philippe H (2008) Bayesian comparisons of codon substitution models. Genetics 180(3):1579–1591, DOI 10.1534/genetics.108.092254

Rubin D (1984) Bayesianly Justifiable and Relevant Frequency Calculations for the Applied Statistician. Ann Stat 12(4):1151 – 1172, DOI 10.1214/aos/1176346785

Talts S, Betancourt M, Simpson D, Vehtari A, Gelman A (2020) Validating Bayesian Inference Algorithms with Simulation-Based Calibration 1804.06788

Teufel A, Ritchie A, Wilke C, Liberles D (2018) Using the mutation-selection framework to characterize selection on protein sequences. Genes 9(8):409, DOI 10.3390/genes9080409

Toni T, Welch D, Strelkowa N, Ipsen A, Stumpf MP (2008) Approximate bayesian computation scheme for parameter inference and model selection in dynamical systems. J R Soc Interface 6(31):187–202, DOI 10.1098/rsif.2008.0172

Wegmann D, Leuenberger C, Neuenschwander S, Excoffier L (2010) ABCtoolbox: a versatile toolkit for approximate bayesian computations. BMC Bioinformatics 11(1), DOI 10.1186/1471-2105-11-116

Yang Z (1994) Maximum likelihood phylogenetic estimation from DNA sequences with variable rates over sites: Approximate methods. J Mol Evol 39(3):306–314, DOI 10.1007/bf00160154

Yang Z (2007) PAML 4: Phylogenetic Analysis by Maximum Likelihood. Mol Biol Evol 24(8):1586–1591, DOI 10.1093/molbev/msm088

Yang Z, Nielsen R, Goldman N, Pedersen AMK (2000) Codon-substitution models for heterogeneous selection pressure at amino acid sites. Genetics 155(1):431–449, DOI 10.1093/genetics/155.1.431
